# Exploring the demographics of teachers who experience secondary traumatic stress

**DOI:** 10.1186/s40359-023-01217-z

**Published:** 2023-06-15

**Authors:** Ofelia Castro Schepers

**Affiliations:** Curriculum and Instruction, Purdue University, 100 N. University St, Beering Hall Rm 4120, West Lafayette, Indiana 47906 USA

**Keywords:** Secondary traumatic stress, Classroom teachers, Survey, Urban schools

## Abstract

**Background:**

Over the last decade, the prevalence of childhood and adolescent trauma has continued to gain public notice, forcing educational systems to explore the impact of these traumas on students, teachers, and schools. Some have implemented trauma-informed practices that are purported to be effective for supporting students in classrooms. Researchers have explored the possibility of its adverse effect on teachers as secondary traumatic stress. This study aimed to explore Secondary Traumatic Stress (STS) in classroom teachers in one small urban school district. STS is said to capture how professionals working closely with traumatized populations are impacted by witnessing their clients' experiences. This phenomenon has adversely affected attrition in other helping professions yet is only recently the focus of educational research.

**Methods:**

The author administered an attitudinal survey to measure levels of STS in one small, urban school district in the U.S. The population sample in this study mirrored that of the district's population and that of national demographics of teachers in the U.S. Descriptive statistics were used to run regression analysis against the STS data.

**Results:**

The findings showed that most teachers experience STS levels within the normal range. White, working-class elementary school teachers experienced higher levels of STS than their K-12 classroom teacher peers.

**Implications:**

The results support a need to continue research on the impact of STS on teachers. Further investigations could inform teacher preparation programs and professional development to identify practices that can help mitigate STS in teachers.

Helping professionals, such as therapists, social workers, and first responders, have long relied on research and support practices to approach phenomena such as Secondary Traumatic Stress (STS) in their workforces. The field of psychology first began exploring this phenomenon to understand how professionals who work closely with traumatized populations are impacted by bearing witness to their clients' experiences [29]. Recent work [8, 13, 19, 44, 58] has identified a need to consider the effect and possible mitigation of STS in teachers.

Research has documented teacher stress and anxiety as early as the 1800s with the advent of urban public schooling. For example, David Tyack's history of United States urban education discusses a survey administered by the National Education Association in 1905 that shows the numerous and varied stressors experienced by teachers. Beyond presenting a general overview of teachers' concerns ranging from compensation to laws imposed on female teachers' rights to make life decisions without losing their job (e.g., marriage, having children), the survey also captured many expressions of emotional exhaustion, entrapment, stress, and frustration, as shown below:

*"The strain is so great, and the salary allows no sum for recuperation; there is no line of work which so drains that vitality." (Emphasis added)*

*"If I could get something else that would pay better, I would give up teaching in spite of the fact that I love the work, but after teaching for ten years, most of us are unfit for anything else. We give the best years of our lives to work, and how few live to enjoy the pension!" (Emphasis added)*

*"I am so worn out from teaching sixty pupils that most of my money goes for medicine and trips for my health." (Emphasis added)*

*(Tyack, 1974, pp. 258–259).*

Over a century ago, teachers were expressing signs of stress, "drain[ed] … vitality," and health problems directly related to their profession ([63], p. 258). Societal standards have positioned teachers as more caring, loving, and kinder than people outside of the field of education. Not only Widespread public discussion suggests an expectation for teachers to be loving, caring, and empathetic with their students, but teachers find the desire to provide this emotional support through care and love for children as fundamental to their identity as teachers [1, 9, 46, 47, 59]. Fostering these relationships and mutual empathetic engagement in a classroom is necessary for classrooms to thrive as a community. Scholars and educators tout emotional and empathetic engagement as essential to foster a welcoming environment for students in classrooms [10, 61]. Similarly, research has shown that students who can connect with their teachers have more substantial academic and social outcomes [23, 28, 31, 42].

Over the last few years, the prevalence and knowledge of trauma experienced by children have made public headlines [38, 45, 55, 60]. Most recently, the experience of collective trauma caused by the COVID-19 pandemic drastically increased those experiencing trauma and further highlighted disparities in access to mental health support for students as schools closed [25, 51] More than two-thirds of children will have reported experiencing at least one traumatic event by age 16 [54]. Children who experience trauma represent all racial and socio-economic levels, and the concentration of students who experience trauma in schools that receive Title I^1^funding is likely much higher [4, 40].

The Substance Abuse and Mental Health Services Administration (SAMHSA) identifies the following as potentially traumatic events: psychological, physical, or sexual abuse; community or school violence; witnessing or experiencing domestic violence; national disasters or terrorism; commercial sexual exploitation; sudden or violent loss of a loved one; refugee or war experiences; military family-related stressors; physical or sexual assault; neglect; serious accidents or life-threatening illness [54]. Additionally, numerous researchers have identified the impact of experiencing childhood trauma on students' lives. There is a correlation between childhood trauma and a multitude of adverse effects, including a decrease in achievement scores as stress related to trauma increases and higher instances of students being referred to an Individualized Education Program for behavior or learning concerns [15, 30, 41, 48–50, 52, 62]. For the scope of this work, the definition of trauma has been expanded to include insidious trauma. Insidious trauma encompasses trauma caused by systemic inequalities and is used by researchers in critical trauma studies. Researchers have been aiming to understand the psychological consequences of racism in recent work [16, 17, 20, 21, 27, 36]. This definition captures experiences in the student population that many students endure and, importantly, captures traumas that schools and teachers within the system cause. This type of trauma captures "little t" traumas, the adverse effects of poverty, discrimination, racial trauma, generational trauma, etc. Unfortunately, widely used definitions do not encompass these types of traumas.

Schools have begun to address traumatic events' impact on students by implementing trauma-informed practices in schools and increasing support for those delivering this care is on the rise [3, 43]. Exposure to witnessing or hearing other people's trauma can create a secondary traumatic stress reaction for those supporting children who have experienced traumatic events [8, 13, 19, 29]. This positions teachers uniquely, as primary adults in children's lives, spending roughly eight hours a day, five days a week, for 180 days a year with them, they are able to get to know their students deeply, which can include exposing themselves to the prolonged witnessing of student trauma [44]. Further, research has documented how school personnel perceives themselves as incompetent in supporting students who experience traumatic stress [2, 3, 22].

The focus of previous studies on teacher stressors has been on the impact of school organizational factors such as policy implementation and teacher requirements [34, 37, 39, 53, 57] on teacher stress. While other studies have explored stress that impacts teachers directly, this study explores secondary traumatic stress because it pays particular attention to the stress caused by hearing about students' trauma. Given that secondary traumatic stress has the potential to generate high levels of burnout in other helping professions [29], it seemed pertinent to explore how it impacts teachers and the demographics of those most highly impacted.

Understanding STS from its research-based origin helps understand how the research can bridge it into a profession that has rarely been addressed in STS studies. STS studies are primarily conducted in psychology and other helping professions (i.e., nursing, social work, etc.). These studies don't apply directly to the field of education because the contexts in which they were conducted vary drastically from the organization of schools. Because of its grounding in psychology, studies use measures that directly reference a client-patient relationship [14]. Once researchers identify a need to be concerned about secondary traumatic stress, they can implement various coping mechanisms that are not only individually focused but also focused on organizational shifts, such as spending less time with certain clients, shifting the workload to other providers, adjusting time in first respondent positions, etc. [18]. In schools, some methods, such as spending less time with clients or shifting positions to not be on the first line of defense as a first responder, are not feasible in schooling environments and may cause more harm to students than good. This study was developed to better understand what teachers experience STS and to what extent, allowing further work to focus on mitigating these factors through individual and systematic shifts in schools, districts, and teacher preparation programs specific to the population's needs.

## Methods

### Context

This study took place in a small, urban district, Franklin,^2^ in the High Plains of the United States, where the majority of the students in the district are Latinx and qualify for Free and Reduced Lunch. The district has 12 schools, seven primary schools, two intermediate schools, two middle schools, and one high school. The population's demographics have remained constant over the last five years, as seen in Table 1.

**Table 1:** Student demographics of Franklin School District

Total Enrollment	5,021
Racial/Ethnic Makeup	76.9% Hispanic 15.9% White 2.14% Black or African American 4.99% Other
English Language Learners	62.3%
Migrant	12.8%
Free/Reduced Lunch	78.9%

(Franklin School District, 2017)

The demographics of this district made this school district an ideal site because it resembled other districts nationwide in terms of teacher make-up (race, gender, SES), and its student demographics resemble that of larger urban settings as well. Table 2 has the teacher demographics of the district. The district has been implementing initiatives to increase its teacher diversity. Still, it has failed to make the gains other districts make in recruiting and retaining minority teachers in neighboring districts.

**Table 2:** Certified staff demographics of Franklin School District

Total Employed Teachers	377	
Sex Assigned at Birth	27.8%	Male
	72.2%	Female
Racial/Ethnic Makeup	9.9% 83.4%	Hispanic White
	0.8%	Asian
	4.3%	Black or African American
	1.3%	Two or more Races

Data gathered from School Officials of Franklin School District. (Franklin School District, 2017)

### Procedure

A web-based survey was distributed to the district e-mail of the 377 classroom teachers employed by the Franklin School District in January 2017 to examine secondary traumatic stress (STS) in teachers. All certified teachers in the urban public school district in the High Plains region of the United States had an equal opportunity to participate in the survey. With approval from the school district, an e-mail was sent to every classroom teacher employed in the district. This excluded interventionists, administrators, and other staff who work with children on an as-needed basis. Upon giving electronic consent, participants proceeded to the twenty-eight Likert-style survey questions and accompanying demographic information. The survey data was collected on the online survey tool Qualtrics. The survey was open for one month, with a reminder sent after two weeks. The survey was closed at the end of the month, and the data was used for analysis.

These research questions guided the study:

- What level of STS is evident in K-12 public school teachers?
  - ◦ Does it vary by school demographics?
  - ◦ Does it vary by teacher demographics?

### Participants

The demographics of the study are shown in Table 3. The demographics are broken down by sex and race/ethnicity. Because the survey drew from a closed population sample, the population demographic is also displayed in Table 3. A total of 377 surveys were e-mailed out, and 115 complete surveys were returned.

**Table 3:** Demographics of teacher population and survey sample percentages

	**Population**	**Survey Sample**
**N**^**a**^	377	115
Gender		
Female	72.2	73
Male	27.8	27
Racial/Ethnic Makeup		
White	83.4	79.1
Hispanic/Latino	9.9	11.3
Black/African American	4.3	2.6
Native American	0	4.3
Asian	0.8	1.7
Other	1.3	1.7

^a^Numbers in all rows except the 2^nd^ one are represented in percentage

The demographics of the survey sample are representative of the Franklin teacher population. This representation can be attributed to the randomization of the survey data collection. There was a 31% response rate which is a reasonable response rate for an outside e-mail survey, and it is typical for external surveys to get a 10–15% response rate [32]. Not only was the survey sample representative of the population, but it was also similar to national averages in the profession. Specifically, in this state, male and female demographics are 23% and 77%, respectively, according to national data [56].

### Instrument

This study used a published, validated measure. The "Teacher Secondary Traumatic Stress Scale" was developed based on a need to create a measure specific to the field of education. It is a twenty-eight Likert-style measure. The publication of the instrument was completed in the appendix of [8], in which the instrument was used and showed validity and reliability. The appendix included the pilot study data and validation procedures. Further, the exhaustive tool validation can be seen in a published dissertation [7]. The survey instrument was validated via a pilot study conducted in the fall of 2015 and has been used in other published [8] and unpublished works, such as dissertations and theses. The survey drew from STS studies in the field of psychology [14] and adapted these to education by making the wording more appropriate for educators. Further, the author developed a construct measure using the symptoms of secondary traumatic stress. The Cronbach's alpha during the pilot was 0.92, which is high for an attitudinal test and showed internal consistency. With good validity and reliability, it is suitable for the assessment of secondary traumatic stress among educators in a K-12 context.

The Teacher Secondary Traumatic Stress Scale was developed to capture how teachers take on others' traumas, specifically their students' traumas. I chose to conceptualize STS as "embodiment" and connect it closely to this theory as posited by the critical feminist underpinnings of Anzaldua [5, 11, 12, 26]. Embodiment, as is used by these theorists, considers historical oppression and its imprint on those that are oppressed, as well as the societal factors that have historically impacted certain communities. Importantly for this study, the concept of embodiment indicates that these historical and systemic traumas are felt in the bodies of teachers. That is to say, they can have an impact on the physical, emotional, and mental aspects of a person, and thus the body should be a place where one can theorize about the effects of STS. The quantitative tool of the Teacher Secondary Traumatic Stress Scale attempts to capture a phenomenon that can be theorized in ways that vary from how STS is typically seen in the field of psychology: an individualized problem to fix. The survey captured the witnessing and embodiment of student traumas that teachers take on, but these critical traumas allow the author to move away from pathologizing and individualizing.

The attitudinal survey used four attitudinal scoring measures that were intended to capture STS, or, as conceptualized, embodiment. Respondents were able to respond to the items using the four attitudinal responses of Never, Occasionally, Very Often, or Always, based on how much they agreed with the statements that were made. Demographic variables were added to the end of the survey as separate questions.

### Data analysis

Analysis began by cleaning the data. This included dropping incomplete cases and reverse coding the positively worded items so that they would be scored the same way as the negatively worded questions.

To answer the first question, "What level of STS is evident in K-12 public school teachers?" the level of STS for each respondent was calculated; this was done by calculating the total theta of each respondent. To do this, the categories were collapsed from a four-point scale to a 2-point scale. The Rasch Model was used and is based on the idea that a person's probability of answering correctly for any given question will depend on the person's ability (θ_p_) and the difficulty of the question itself (b_i_). This can be seen in the equation Pp,bi = exp⁡(p-bi)1 + exp⁡(p-bi). In this case, a person's probability of agreeing to any given question will depend on the person's level of STS (θ_p_) and the difficulty of the question itself (*b*_*i*_). After the respondents were assigned a theta, their theta was compared to the item difficulties using a Wright Map. The Wright Map provided a representation of the administered survey by placing the difficulty of the items on the same measurement scale as the construct level of respondents (theta).

Having calculated the theta for all respondents, descriptive and inferential statistics were analyzed for the respondents. The descriptive statistics provided the information needed to respond to the sub-questions of question number one: "Did STS vary by school and teacher demographics?" This question was answered using regression analysis of the respondents' demographics in the survey. I began by running a regression on STS by teacher ethnicity to determine a baseline for the intercept of the statistical model. A regression on "school type," holding all other variables constant, was then calculated. Similar regressions of STS on teacher demographics holding all variables constant were then calculated.

The final step of the survey analysis was to ensure the test's validity and reliability. Though this was done through an earlier pilot study, I wanted to ensure that the survey held robust reliability statistics with the changes that were made after the pilot test analysis. This analysis was completed using classical test theory and checking item average, difficulty, and correlation for reliability. A standard error of measurement was also performed.

## Results

The findings indicated that teachers in Franklin School District all experienced STS at varying levels. Some teachers had lower levels of STS, while others had higher levels of STS. Overall, many of the teachers experienced moderate levels of STS, with the lowest raw score of about eight points out of zero. Although one person experienced STS at very low levels because the majority of their responses were not aligned with positive responses or "Agree," there was not a single participant that experienced no STS. 

The descriptive statistics were followed by inferential statistics intended to better understand how the items move on the constructed scale. The items were all correlated, aside from two positively worded items. The positively worded items ensured that participants read all questions. The Cronbach's alpha of 0.81 indicated that the survey had a moderately high level of reliability which is slightly lower than the pilot study's 0.95. Still, the sample changed to include a more heterogeneous population than the pilot study.

The sub-question was analyzed using inferential statistics. These statistics indicated that Elementary teachers experienced higher levels of STS than that Middle School and High School teachers. The difference was statistically significant for High School teachers compared to Elementary School teachers. This analysis showed that White, working-class teachers experience higher levels of STS than middle-class teachers and those who identify as non-White. Also, upper-middle-class teachers experienced far lower levels of STS than their middle-class and working-class peers.

### Question 1: What level of STS is evident in K-12 public school teachers?

**Fig. 1:**
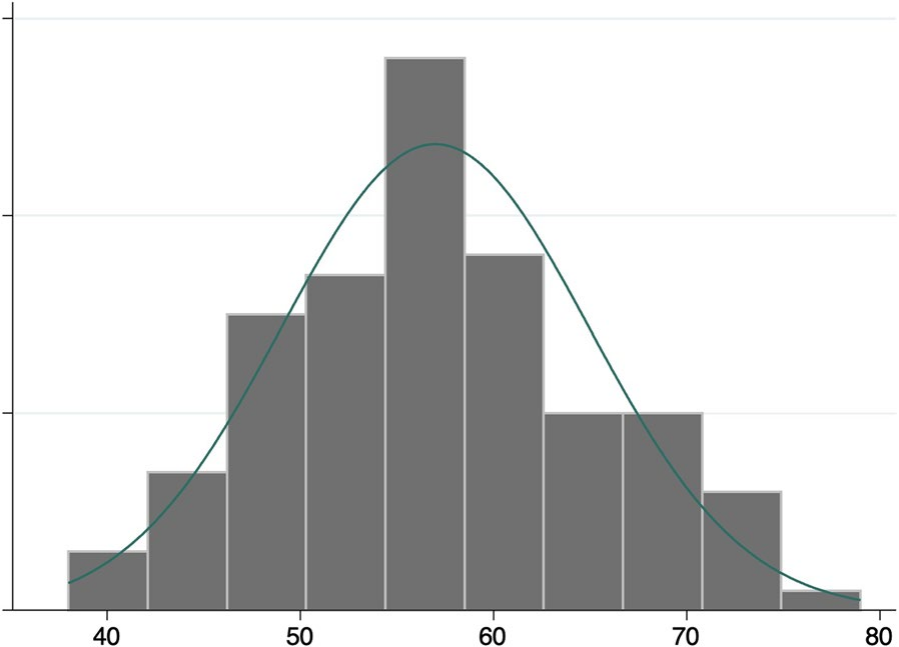
Histogram of Teachers' Total STS Scores

The scores are normal distribution, with an average of 57 and a standard deviation of 7.9. The responses had a minimum total score of 38 and a maximum total score of 79, with the overall possibility minimum and maximum ranging from 0–112. This indicates that teachers are experiencing levels of STS at varying levels. In other words, teachers embody the traumas of students at different rates but overall regular occurrences. The normal curve shows that there are teachers who experience low levels of STS and some who experience high levels of STS. However, the bulk of teachers indicated through their responses that they experienced moderate levels of STS, as seen in Fig. 1, where the majority of the distribution is in the middle of the histogram. The minimum and maximum show that there were zero teachers that experienced STS zero, but also zero teachers that experienced the max level of STS that would be possible.

#### Sub Question 1.a.: Does [STS] vary by school demographics?

To answer the sub-question, "Does this vary by school demographics?" the level of STS for all teachers was calculated and ran a regression of the variables that held constant school demographics. Table 4 has the regression outcomes for levels of STS by school demographics. The intercept represents Elementary School Teachers.

**Table 4:** Secondary traumatic stress of teachers by school demographics

	**Estimated**	**Standard Error**	**T-Value**	**Pr(> [t])**
Intercept	31.21	2.13	14.67	< 2e-16***
Middle School	-0.91	3.42	-0.26	0.79
High School	-5.88	3.29	-1.79	0.08

*N* = *115;* Significance: 0^(***)^ 0.001^(**)^ 0.01^(*)^ 0.05^(.)^

The results show that elementary school teachers experience greater levels of secondary traumatic stress than both middle school and high school teachers. However, this difference is only significant when considering high school teachers, and the level of significance nears the 0.05 level.

#### Sub Question 1.b.: Does [STS] vary by teacher demographics?

To answer the sub-question, "Does this vary by teacher demographics?" I calculated the level of STS for all teachers, the theta, and ran a regression of the variables that held constant all teacher demographics reported. Table 5 has the regression outcomes for levels of STS by teacher demographics. The intercept represents White, Female, Elementary school teachers who consider themselves as having been brought up in a middle-class family and are in the middle of their career. This was chosen as this is the typical demographic of a practicing teacher [33].

**Table 5:** Levels of secondary traumatic stress in teachers by teacher demographic

	**Estimated**	**Standard Er**	**T-Value**	**Pr(> [t])**
Intercept	31.77	2.55	12.46	< 2e-16***
Working Class	6.01	2.63	2.29	0.02 *
Upper Middle Class	-8.52	4.16	-2.08	0.04 *
Male	-2.72	2.66	-1.02	0.34
Beginning Teacher	-1.64	2.87	-0.57	0.57
Experienced Teacher	-4.81	3.01	-1.59	0.11
Non-White	-7.13	3.17	-2.25	0.03 *

*N* = *115;* Significance: 0^(***)^ 0.001^(**)^ 0.01^(*)^ 0.05^(.)^

Table 5 shows that Class and Race/Ethnicity have statistically significant differences in how they experience levels of secondary traumatic stress. Teachers who grew up in working-class and upper-middle-class families experience STS at different levels than those who grew up in middle-class families. Furthermore, teachers from a working-class background experienced STS levels at the highest rates, middle-class backgrounds experienced it at the next highest rate, and teachers from upper-middle-class backgrounds experienced STS at lower rates than teachers from a middle-class background. These findings were significant between the 0.01 and 0.05 significance levels.

How teachers experienced levels of STS also varied by race/ethnicity at a statistically significant level. Teachers who identified as non-White experience lower levels of STS than their White counterparts. This was significant at the 0.03 level. Though male, beginning, and experienced teachers experienced lower levels of STS than the intercept described above, none of these variables were statistically significant, and their numbers could have been caused by chance.

## Discussion

Over the last decade, studies have slowly taken up the concept of secondary traumatic stress in educators in parallel with the implementation of trauma-informed practices in schools for students. These studies have explored various populations, most recently looking at STS in relation to the utilization of trauma-informed care practices in educational settings [58]. This study is one of few to solely explore STS in classroom teachers with the intention of understanding who in the population was more likely to experience higher levels of STS. This is intended to add to the literature of our understanding of STS in educational spaces. The study collected data from a school district that had not been implementing any specific trauma-informed care or practices. The district was largely comprised of Title I schools. Though comparisons across measures differ, the results of this study were lower than those found by Borntrager et al. [13]. However, they were comparable to the STS baseline means reported by Sprang & Garcia [58] and Christian-Brandt et al. [24].

Findings in this study indicated that the levels of STS were shockingly average. However, because of the representative sample, findings identified specific demographic factors of teachers that experienced higher levels of STS. White, working-class elementary teachers experienced elevated levels of STS than non-White, working-class high school teachers at statistically significant levels.

Possible reasons for white, working-class elementary teachers experiencing higher levels of STS are noted in work around the white savior complex [6, 35]. This work situates the individual behavior in the larger context of a largely non-white, low-socioeconomic student population that attend the Franklin School District and narratives of a need to save those who are viewed as less fortunate. Further qualitative data (not included in this paper) expands on more nuanced explanations of why this population experiences higher levels of STS that delve into the white savior complex language used among participants of the larger study, which included interviews. When considering the Chicana feminist frameworks, a need to save others is not aligned with this work. It thus can potentially explain the already embodied historical traumas and personal experience that allows Teachers of Color to detach the individual and situate it in oppressive systems [5, 11, 26]. Meaning, Teachers of Color have experienced systems that are oppressive and do not see children in need of saving,rather, they see broken systems in need of fixing. Further, taking on a savior mentality reifies some of the main symptoms of STS to perpetually work in opposition to lower STS, higher levels of intrusion, and arousal when teachers think about saving children.

This study is deeply significant in that it indicates that though there is a normal curve on how many people experience STS, those who are most highly impacted are a population that makes up roughly 80% of the teacher workforce in this district. There is a continued need to focus on STS in teaching because, in other helping professions, STS has been linked to burnout and turnover. The benefit of identifying levels of STS could lead to the creation of resources to help mitigate the symptoms at the individual and school levels and lean on studies that are pursuing mitigation efforts such as trauma-informed care utilization [58] and trauma-informed practices in higher education for preservice educators [8]. Moreover, additional longitudinal research is needed to fully understand if STS impacts attrition rates in schools in addition to the work focused on intent to leave [24].

### Limitations

As with any study that utilizes survey methodology, voluntary participation, and self-selection bias are always of concern. However, the relatively high response rate of 35% for an external survey and the sample being representative of the population suggests that this was moderately accounted for. Additional concerns with survey data include the self-reporting of STS. Anonymizing the collection methods was designed to ease concerns of self-reporting bias. Future research would be strengthened by using additional measures to account for other possibly confounding variables, such as primary trauma, burnout, school climate inventories, and other constructs that align closely with secondary traumatic stress. The next steps using this dataset would be to investigate each of the areas that account for STS and further interactions between STS and mitigating factors.

## Data Availability

The datasets used and/or analyzed in the current study are available from the corresponding author upon reasonable request.

## Footnotes

1 Title I funding from the U.S. government provides financial assistance to local educational agencies and schools with high numbers or high percentages of children from low-income families. Federal funds are currently allocated through four statutory formulas that are based primarily on census poverty estimates and the cost of education in each state https://www2.ed.gov/programs/titleiparta/index.html

2 Pseudonym.
